# Obituary

**Published:** 1910-01-22

**Authors:** 


### Obituary.

The death took place on January 10 of Mr. Septimus J. Lee, L.R.C.P. (Lond.), M.R.C.S. (Eng.), of Brampton, Cumberland. He had been resident medical officer at the Hospital for Epilepsy and Paralysis, Maida Vale, since April 1909. Mr. Lee studied at Edinburgh and at Middlesex Hospital, and was appointed to the Hospital for Epilepsy as soon as he had qualified last year.

